# Novel nomogram for predicting survival in advanced non-small cell lung cancer receiving anti-PD-1 plus chemotherapy with or without antiangiogenic therapy

**DOI:** 10.3389/fimmu.2023.1297188

**Published:** 2023-11-08

**Authors:** Yahua Wu, Chengliu Lv, Mingqian Lin, Yaping Hong, Bin Du, Na Yao, Yingjiao Zhu, Xiaohui Ji, Jiancheng Li, Jinhuo Lai

**Affiliations:** ^1^ Department of Medical Oncology, Fujian Medical University Union Hospital, Fuzhou, China; ^2^ Department of Radiation Oncology, Fujian Medical University Cancer Hospital, Fuzhou, China; ^3^ Department of Medical Oncology, Chongqing University Cancer Hospital, Chongqing, China

**Keywords:** advanced NSCLC, anti-PD-1, combined therapy, nomogram, survival

## Abstract

**Background:**

This study aimed to develop and validate a novel nomogram to predict survival in advanced non-small cell lung cancer (NSCLC) receiving programmed cell death 1 (PD-1) inhibitor plus chemotherapy with or without antiangiogenic therapy.

**Methods:**

A total of 271 patients with advanced NSCLC who received anti-PD-1 plus chemotherapy with or without antiangiogenic therapy were enrolled in our center and randomized into the training cohort (*n* = 133) and the internal validation cohort (*n* = 138). Forty-five patients from another center were included as an independent external validation cohort. The nomogram was created based on the multivariate Cox regression analysis to predict overall survival (OS) and progression-free survival (PFS). The performance of the nomogram was assessed using the concordance index (C-index), the time-dependent area under the receiver operating (ROC) curves (AUCs), calibration curves, and decision curve analysis (DCA).

**Results:**

Four factors significantly associated with OS were utilized to create a nomogram to predict OS: Eastern Cooperative Oncology Group performance status (ECOG PS), programmed cell death-ligand 1 (PD-L1) expression, chemotherapy cycle, and pretreatment lactate dehydrogenase–albumin ratio (LAR). Six variables significantly associated with PFS were incorporated into the development of a nomogram for predicting PFS: ECOG PS, histology, PD-L1 expression, chemotherapy cycle, pretreatment platelet to lymphocyte (PLR), and pretreatment LAR. The C-indexes of the nomogram for predicting OS and PFS were 0.750 and 0.747, respectively. The AUCs for predicting the 6-month, 12-month, and 18-month OS and PFS were 0.847, 0.791, and 0.776 and 0.810, 0.787, and 0.861, respectively. The calibration curves demonstrated a good agreement between predictions and actual observations. The DCA curves indicated that the nomograms had good net benefits. Furthermore, the nomogram model was well-validated in the internal and external cohorts.

**Conclusion:**

The novel nomogram for predicting the prognosis of advanced NSCLC receiving anti-PD-1 plus chemotherapy with or without antiangiogenic therapy may help guide clinical treatment decisions.

## 1 Introduction

Lung cancer is a widespread and fatal malignancy worldwide (1). Non-small cell lung cancer (NSCLC) accounts for 85% of lung cancer (2). Most patients are diagnosed at an advanced stage. Platinum-based chemotherapy is the standard first-line treatment regimen for advanced NSCLC patients without driver mutations (3). However, the efficacy of chemotherapy alone is limited, with response rates ranging from 25% to 35% (4).

Immune checkpoint inhibitors (ICIs) such as programmed cell death 1 (PD-1) inhibitors have demonstrated significant advantages in antitumor therapy and are widely used in clinical practice (5). Several studies have shown that ICIs improve the prognosis of patients with advanced NSCLC in comparison to chemotherapy (6–9). However, it has been reported that only approximately 20% of patients exhibit a favorable response to ICIs (10, 11). Therefore, the combination therapy has attracted the attention of clinicians. An increasing body of evidence suggests that immunotherapy and chemotherapy have a synergistic effect. Firstly, chemotherapy enhances the sensitivity of tumor cells to immune-mediated killing (12). Additionally, chemotherapy increases the immunogenicity of tumor cells and eliminates immunosuppressive cells (13, 14). Moreover, immunotherapy has the potential to enhance patients’ sensitivity to chemotherapy. The KEYNOTE-189 study analyzed the efficacy of pembrolizumab plus chemotherapy versus chemotherapy alone in patients with non-squamous NSCLC, and the results suggested that pembrolizumab combined with platinum-based chemotherapy significantly prolonged overall survival (OS) and progression-free survival (PFS) when compared with chemotherapy alone (6). The KEYNOTE-407 study also demonstrated a significant improvement in OS and PFS for patients with metastatic squamous NSCLC receiving pembrolizumab plus chemotherapy (15).

Combination immunotherapy is increasingly recommended as a first-line treatment regimen for advanced NSCLC (16). However, identifying the optimal population for combination immunotherapy remains a significant challenge. Programmed cell death-ligand 1 (PD-L1) expression is now commonly used as a predictor of immunotherapy efficacy in NSCLC. However, PD-L1 expression alone is not sufficient to predict efficacy accurately (17), especially in the modality of combination immunotherapy. As the combination immunotherapy approach is widely applied in clinical practice, it is necessary to explore more potential indicators to predict survival. Therefore, in this study, we aim to identify predictive markers that influence efficacy and attempt to create a novel nomogram predicting the prognosis of advanced NSCLC patients who were treated with anti-PD-1 plus chemotherapy with or without antiangiogenic therapy, which is beneficial for guiding clinical treatment decisions.

## 2 Methods

### 2.1 Study population

This retrospective study included 316 patients with advanced NSCLC receiving anti-PD-1 plus chemotherapy with or without antiangiogenic therapy from two centers: 271 patients from our center between January 2018 and January 2023 and 45 patients from another center between January 2021 and March 2023. The inclusion criteria were as follows: 1) pathologically confirmed NSCLC, 2) diagnosis with stages IIIB–IV, 3) age ≥18 years, and 4) Eastern Cooperative Oncology Group performance status (ECOG PS) 0–2. The exclusion criteria were as follows: 1) had EGFR/ALK/ROS1 mutations, 2) had infection and steroid hormone treatment within 1 month prior to treatment initiation, 3) had other sites of primary malignancy, and 4) previously received other antitumor treatments.

### 2.2 Data collection

The clinical information included age, gender, smoking history, Eastern Cooperative ECOG PS, histology, PD-L1 expression, clinical stage, brain metastases, bone metastases, liver metastases, chemotherapy cycle, antiangiogenic therapy, and hematological biomarkers including neutrophil to lymphocyte ratio (NLR), platelet to lymphocyte (PLR), monocyte to lymphocyte ratio (MLR), and lactate dehydrogenase–albumin ratio (LAR). Peripheral blood biomarkers were collected from 1 week before the initiation of anti-PD-1 treatment. The NLR, PLR, MLR, and LAR were calculated as follows: NLR = absolute neutrophil count (ANC)/absolute lymphocyte count (ALC), PLR = platelet count/ALC, MLR = absolute monocyte count (AMC)/ALC, and LAR = lactate dehydrogenase (LDH)/albumin (Alb).

### 2.3 Treatment

All patients received first-line anti-PD-1 therapy in combination with chemotherapy. The PD-1 inhibitors included pembrolizumab, sintilimab, camrelizumab, and tislelizumab. Chemotherapy regimens included PP (pemetrexed + cisplatinum/carboplatin) and TP (paclitaxel + cisplatinum/carboplatin). Anti-angiogenic agents included bevacizumab and endostar. All patients received at least two cycles of anti-PD-1 plus chemotherapy.

### 2.4 Outcomes

The primary outcome was overall survival (OS) and progression-free survival (PFS). OS was measured from the time of first treatment with PD-1 inhibitors until death due to any cause. PFS was calculated from the date of first treatment with PD-1 inhibitors until disease progression or death by any cause. The secondary outcome was immunotherapy response, including objective response rate (ORR) and disease control rate (DCR). ORR was the proportion of complete response (CR) or partial response (PR). DCR was the proportion of CR or PR or stable disease (SD). The iRECIST criteria were used to assess tumor treatment response (18). The evaluation of the efficacy of tumor treatment was performed independently by two experienced clinicians. When there were disagreements between two clinicians, another experienced clinician was invited to participate in the efficacy assessment. The follow-up was conducted through an electronic medical record system and telephone. The last follow-up was conducted in June 2023. The median follow-up time for the overall population was 24 months.

### 2.5 Nomogram

Eligible patients at our center were randomly assigned to either the training cohort (*n* = 133) or the internal validation cohort (*n* = 138). The training cohort was used to identify prognostic factors and construct the nomogram model, while the internal validation cohort was used to validate the performance of the nomogram model. Additionally, we included an external validation cohort consisting of 45 patients from another center. Furthermore, we utilized the concordance index (C-index), the time-dependent area under the receiver operating characteristic (ROC) curves (AUCs), calibration curves, and decision curve analysis (DCA) to assess the performance of the nomogram model.

### 2.6 Statistical analysis

Statistical analyses were performed using SPSS software version 25.0 and R version 4.2.1. Quantitative data was presented using the median (interquartile range, IQR). Categorical variables were analyzed by the chi-square test or the Fisher’s exact test, and numerical variables were analyzed by the Kruskal–Wallis test. Kaplan–Meier curves and log-rank test were utilized to analyze OS and PFS. Univariate Cox analyses was performed for each variable, and variables that were statistically significant (*P* < 0.05) were included in the multivariate Cox analyses to identify independent prognostic factors affecting OS and PFS. Univariate and multivariate logistic regression analyses were used to identify factors that independently influenced the ORR. The results were considered statistically significant when the two-sided *P*-value was less than 0.05.

## 3 Results

### 3.1 Patients’ characteristics

A total of 316 patients with advanced NSCLC who received anti-PD-1 plus chemotherapy with or without antiangiogenic therapy were included in this study. Among them, 184 (58.2%) were younger than 65 years, 266 (84.2%) were men, and 226 (71.5%) had smoking history. The majority of patients (88.0%) had an ECOG PS of 0–1. According to PD-L1 expression, patients with low PD-L1 expression (1%–49%) accounted for 24.4%, and those with high PD-L1 expression (≥50%) accounted for 15.8%. There were 271 eligible patients in our hospital, of whom 133 and 138 were randomly assigned to the training cohort and the internal validation cohort, respectively. We also included 45 patients from another hospital as an external independent validation cohort. The baseline clinical characteristics are listed in **Table 1**. No statistically significant differences in baseline characteristics were observed in the different cohorts.

**Table 1:** Baseline characteristics.

Characteristic		All cohorts [cases (%)]	Training cohort [cases (%)]	Internal validation [cases (%)]	External validation [cases (%)]	*P*-value
Total		316	133	138	45	
Age	<65	184 (58.2)	73 (54.9)	81 (58.7)	30 (66.7)	0.585
	≥65	132 (41.8)	60 (45.1)	57 (41.3)	15 (33.3)	
Gender	Female	50 (15.8)	16 (12)	25 (18.1)	9 (20.0)	0.463
	Male	266 (84.2)	117 (88)	113 (81.9)	36 (80.0)	
Smoking history	No	90 (28.5)	34 (25.6)	43 (31.2)	13 (28.9)	0.790
	Yes	226 (71.5)	99 (74.4)	95 (68.8)	32 (71.1)	
ECOG PS	0–1	278 (88.0)	118 (88.7)	117 (84.8)	43 (95.6)	0.279
	2	38 (12.0)	15 (11.3)	21 (15.2)	2 (4.4)	
Histology	Squamous	144 (45.6)	60 (45.1)	66 (47.8)	18 (40.0)	0.429
	Adenocarcinoma	150 (47.5)	62 (46.6)	61 (44.2)	27 (60.0)	
	Others	22 (7.0)	11 (8.3)	11 (8.0)	0 (0.0)	
PD-L1 expression	Negative	59 (18.7)	25 (18.8)	30 (21.7)	4 (8.9)	0.228
	1%–49%	77 (24.4)	32 (24.1)	33 (23.9)	12 (26.7)	
	≥50%	50 (15.8)	18 (13.5)	18 (13.0)	14 (31.1)	
	Unknown	130 (41.1)	58 (43.6)	57 (41.3)	15 (33.3)	
Clinical stage	IIIB–C	22 (7.0)	12 (9.0)	7 (5.1)	3 (6.7)	0.498
	IVA	217 (68.7)	93 (69.9)	98 (71.0)	26 (57.8)	
	IVB	77 (24.4)	28 (21.1)	33 (23.9)	16 (35.6)	
Brain metastasis	No	287 (90.8)	119 (89.5)	128 (92.8)	40 (88.9)	0.775
	Yes	29 (9.2)	14 (10.5)	10 (7.2)	5 (11.1)	
Bone metastasis	No	242 (76.6)	119 (89.5)	128 (92.8)	31 (68.9)	0.439
	Yes	74 (23.4)	14 (10.5)	10 (7.2)	14 (31.1)	
Liver metastasis	No	292 (92.4)	124 (93.2)	126 (91.3)	42 (93.3)	0.935
	Yes	24 (7.6)	9 (6.8)	12 (8.7)	3 (6.7)	
Chemotherapy cycle	2–3	58 (18.4)	23 (17.3)	21 (15.2)	14 (31.1)	0.117
	≥4	258 (81.6)	110 (82.7)	117 (84.8)	31 (68.9)	
Antiangiogenic therapy	No	193 (61.1)	87 (65.4)	79 (57.2)	27 (60.0)	0.381
	Yes	123 (38.9)	46 (34.6)	59 (42.8)	18 (40.0)	
Pretreatment NLR	Median (IQR)	3.1 (1.9, 5.1)	3.0 (1.9, 4.9)	3.2 (1.9, 5.0)	3.4 (2.2, 5.5)	0.520
Pretreatment PLR	Median (IQR)	167.2 (118.2, 240.7)	166.5 (121.4, 233.3)	166.6 (119.5, 238.3)	176.2 (107.8, 248.3)	0.993
Pretreatment MLR	Median (IQR)	0.34 (0.24, 0.48)	0.31 (0.22, 0.45)	0.35 (0.26, 0.48)	0.38 (0.24, 0.60)	0.213
Pretreatment LAR	Median (IQR)	5.6 (4.7, 6.9)	5.3 (4.7, 6.6)	5.7 (4.7, 7.0)	5.5 (4.7, 6.9)	0.837

### 3.2 Efficacy and survival analysis

**Figure 1:**
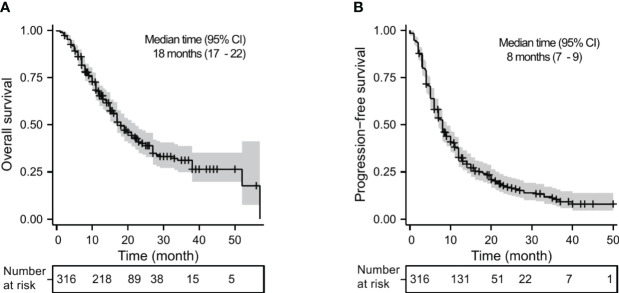
Kaplan–Meier analysis for OS **(A)** and PFS **(B)** in advanced NSCLC receiving PD-1 inhibitor plus chemotherapy with or without antiangiogenic therapy in the whole cohort.

Of the 316 patients, 12 (3.8%) achieved CR, 111 (35.1%) achieved PR, 157 (49.7%) had SD, and 36 (11.4%) had PD. The ORR and DCR were 38.9% and 88.6%, respectively (**Table 2**). A total of 174 (55.1%) patients died with a median OS of 18 months (95% CI, 17–22 months) (**Figure 1A**). The 1-year, 2-year, and 3-year OS rates were 65.2%, 40.3%, and 31.2%, respectively. Two hundred fifty-six patients (81.0%) had disease progression with a median PFS of 8 months (95% CI, 7–9 months) (**Figure 1B**). The 1-year, 2-year, and 3-year PFS rates were 32.6%, 17.0%, and 10.3%, respectively.

**Table 2:** Evaluation of efficacy.

Parameter		*N* (%)
Overall best response	CR	12 (3.8 %)
	PR	111 (35.1%)
	SD	157 (49.7%)
	PD	36 (11.4%)
ORR	CR+PR	123 (38.9%)
DCR	CR+PR+SD	280 (88.6%)

### 3.3 Univariate and multivariate Cox analyses for OS

To determine the effect of different variables on OS, we performed univariate and multivariate Cox analyses in the training cohort (**Table 3**). Univariate Cox analysis showed that ECOG PS, PD-L1 expression, chemotherapy cycle, and pretreatment LAR were significantly associated with OS (all *P* < 0.05). The results of multivariate Cox analysis demonstrated that ECOG PS 2 (HR, 3.807; 95% CI, 2.032–7.134; *P* < 0.001) and pretreatment LAR (HR, 1.106; 95% CI, 1.052–1.162; *P* < 0.001) were unfavorable independent prognostic factors for OS, while PD-L1 expression (1%–49%: HR, 0.413; 95% CI, 0.202–0.845; *P* = 0.015; ≥50%: HR, 0.185; 95% CI, 0.062–0.555; *P* = 0.003) and chemotherapy cycle ≥4 (HR, 0.319; 95% CI, 0.167–0.607; *P* < 0.001) were favorable independent prognostic factors for OS.

**Table 3:** Univariate and multivariate Cox analyses for overall survival in the training cohort.

Characteristics		Univariate analysis		Multivariate analysis	
		HR (95% CI)	*P*-value	HR (95% CI)	*P*-value
Age	<65	Reference			
	≥65	1.420 (0.872–2.312)	0.159		
Gender	Female	Reference			
	Male	1.190 (0.564–2.510)	0.647		
Smoking history	No	Reference			
	Yes	1.303 (0.749–2.269)	0.349		
ECOG PS	0–1	Reference		Reference	
	2	4.146 (2.260–7.606)	<0.001	3.807 (2.032–7.134)	<0.001
Histology	Squamous	Reference			
	Adenocarcinoma	0.632 (0.374–1.066)	0.085		
	Others	1.537 (0.709–3.332)	0.276		
PD-L1 expression	Negative	Reference		Reference	
	1%–49%	0.507 (0.257–0.999)	0.050	0.413 (0.202–0.845)	0.015
	≥50%	0.181 (0.061–0.539)	0.002	0.185 (0.062–0.555)	0.003
	Unknown	0.581 (0.320–1.054)	0.074	0.602 (0.330–1.098)	0.098
Clinical stage	IIIB–C	Reference			
	IVA	1.585 (0.569–4.412)	0.378		
	IVB	2.750 (0.927–8.161)	0.068		
Brain metastasis	No	Reference			
	Yes	1.705 (0.861–3.375)	0.126		
Bone metastasis	No	Reference			
	Yes	1.190 (0.667–2.125)	0.555		
Liver metastasis	No	Reference			
	Yes	1.711 (0.684–4.277)	0.251		
Chemotherapy cycle	2–3	Reference		Reference	
	≥4	0.359 (0.195–0.659)	0.001	0.319 (0.167–0.607)	<0.001
Antiangiogenic therapy	No	Reference			
	Yes	0.795 (0.474–1.332)	0.383		
Pretreatment NLR		1.025 (0.956–1.099)	0.487		
Pretreatment PLR		1.001 (0.999–1.003)	0.149		
Pretreatment MLR		2.302 (0.782–6.772)	0.130		
Pretreatment LAR		1.115 (1.066–1.166)	<0.001	1.106 (1.052–1.162)	<0.001

### 3.4 Univariate and multivariate Cox analyses for PFS

**Table 4** shows the results of the univariate and multivariate Cox analyses of PFS in the training cohort. In the univariate Cox analysis, ECOG PS, histology, PD-L1 expression, chemotherapy cycle, pretreatment PLR, and pretreatment LAR were significantly associated with PFS (all *P* < 0.05). In the multivariate Cox analysis, PD-L1 expression (1%–49%: HR, 0.427; 95% CI, 0.225–0.811; *P* = 0.009; ≥50%: HR, 0.373; 95% CI, 0.176–0.793; *P* = 0.010), adenocarcinoma (HR, 0.589; 95% CI, 0.387–0.896; *P* = 0.013), and chemotherapy cycles ≥4 (HR, 0.229; 95% CI, 0.136–0.285; *P* = 0.001) were favorable prognostic factors for PFS. In contrast, ECOG PS 2 (HR, 3.802; 95% CI, 2.029–7.125; *P* < 0.001), pretreatment PLR (HR, 1.003; 95% CI, 1.001–1.004; *P* = 0.003), and pretreatment LAR (HR, 1.065; 95% CI, 1.015–1.118; *P* = 0.011) were unfavorable prognostic factors for PFS.

**Table 4:** Univariate and multivariate Cox analyses for progression-free survival in the training cohort.

Characteristics		Univariate analysis		Multivariate analysis	
		HR (95% CI)	*P*-value	HR (95% CI)	*P*-value
Age	<65	Reference			
	≥65	1.077 (0.729–1.591)	0.708		
Gender	Female	Reference			
	Male	1.008 (0.563–1.806)	0.978		
Smoking history	No	Reference			
	Yes	1.330 (0.846–2.092)	0.217		
ECOG PS	0–1	Reference		Reference	
	2	3.147 (1.779–5.568)	<0.001	3.802 (2.029–7.125)	<0.001
Histology	Squamous	Reference		Reference	
	Adenocarcinoma	0.618 (0.410–0.931)	0.021	0.589 (0.387–0.896)	0.013
	Others	0.819 (0.388–1.730)	0.601	0.823 (0.355–1.908)	0.650
PD-L1 expression	Negative	Reference		Reference	
	1%–49%	0.489 (0.269–0.888)	0.019	0.427 (0.225–0.811)	0.009
	≥50%	0.351 (0.169–0.728)	0.005	0.373 (0.176–0.793)	0.010
	Unknown	0.744 (0.447–1.238)	0.255	0.815 (0.482–1.337)	0.444
Clinical stage	IIIB–C	Reference			
	IVA	1.287 (0.620–2.672)	0.498		
	IVB	1.426 (0.627–3.241)	0.397		
Brain metastasis	No	Reference			
	Yes	1.246 (0.681–2.279)	0.475		
Bone metastasis	No	Reference			
	Yes	0.801 (0.475–1.351)	0.406		
Liver metastasis	No	Reference			
	Yes	1.632 (0.755–3.529)	0.213		
Chemotherapy cycle	2–3	Reference		Reference	
	≥4	0.313 (0.193–0.509)	<0.001	0.229 (0.136–0.285)	0.001
Antiangiogenic therapy	No	Reference			
	Yes	0.783 (0.519–1.183)	0.245		
Pretreatment NLR		1.049 (0.995–1.106)	0.079		
Pretreatment PLR		1.002 (1.000–1.004)	0.016	1.003 (1.001–1.004)	0.003
Pretreatment MLR		2.056 (0.856–4.940)	0.107		
Pretreatment LAR		1.076 (1.031–1.124)	0.001	1.065 (1.015–1.118)	0.011

### 3.5 Factors associated with ORR

Multivariate logistic regression results confirmed that PD-L1 expression and pretreatment LAR were independent predictors of ORR (**Table 5**). Compared with patients with PD-L1-negative tumors, ORR was significantly improved in patients with PD-L1 ≥50% (OR, 5.923; 95% CI, 1.459–24.044; *P* = 0.013). In addition, patients with higher pretreatment LAR had lower ORR (OR, 0.773; 95% CI, 0.612–0.976; *P* = 0.030).

**Table 5:** Univariate and multivariate logistic regression for ORR.

Characteristics		Univariate analysis		Multivariate analysis	
		OR (95% CI)	*P*-value	OR (95% CI)	*P*-value
Age	<65	Reference			
	≥65	0.713 (0.356–1.428)	0.340		
Gender	Female	Reference			
	Male	0.721 (0.253–2.052)	0.539		
Smoking history	No	Reference			
	Yes	0.795 (0.363–1.740)	0.566		
ECOG PS	0–1	Reference			
	2	0.296 (0.079–1.105)	0.070		
Histology	Squamous	Reference			
	Adenocarcinoma	1.406 (0.686–2.882)	0.353		
	Others	0.563 (0.135–2.336)	0.428		
PD-L1 expression	Negative	Reference		Reference	
	1%–49%	2.269 (0.744–6.922)	0.150	2.372 (0.724–7.769)	0.154
	≥50%	6.686 (1.731–25.823)	0.006	5.923 (1.459–24.044)	0.013
	Unknown	1.571 (0.566–4.365)	0.386	1.405 (0.484–4.078)	0.532
Clinical stage	IIIB–C	Reference			
	IVA	0.1875 (0.528–6.658)	0.331		
	IVB	0.800 (0.187–3.423)	0.764		
Brain metastasis	No	Reference			
	Yes	1.380 (0.455–4.184)	0.569		
Bone metastasis	No	Reference			
	Yes	1.028 (0.432–2.447)	0.950		
Liver metastasis	No	Reference			
	Yes	0.358 (0.072–1.795)	0.212		
Chemotherapy cycles	2–3	Reference		Reference	
	≥4	3.228 (1.119–9.308)	0.030	2.987 (0.974–9.156)	0.056
Antiangiogenic therapy	No	Reference			
	Yes	1.040 (0.505–2.139)	0.916		
Pretreatment NLR		0.960 (0.848–1.087)	0.523		
Pretreatment PLR		0.998 (0.995–1.001)	0.305		
Pretreatment MLR		0.671 (0.121–3.725)	0.648		
Pretreatment LAR		0.762 (0.615–0.945)	0.013	0.773 (0.612–0.976)	0.030

### 3.6 Construction of the nomogram

**Figure 2:**
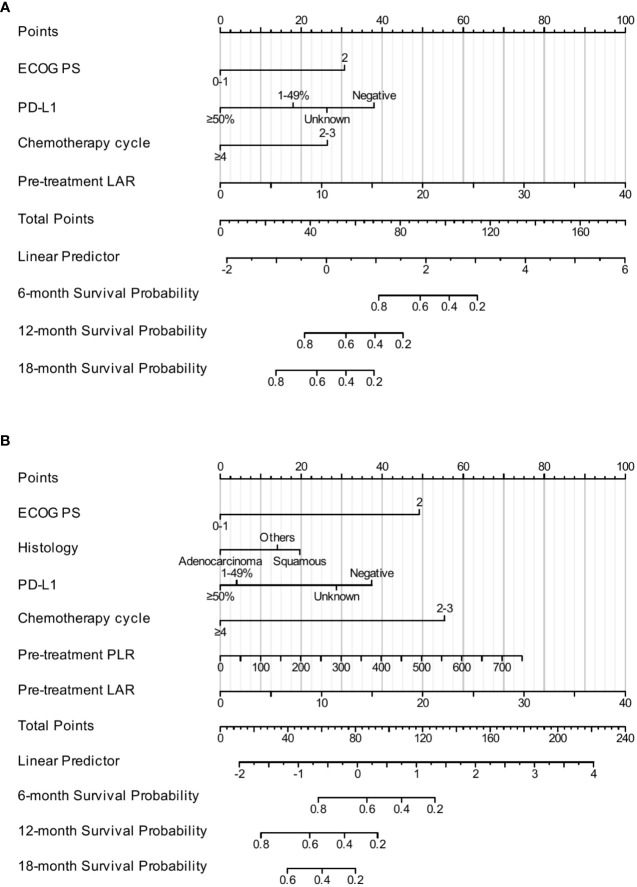
The construction of the nomogram. The nomogram for predicting the 6-month, 12-month, and 18-month OS **(A)**. The nomogram for predicting the 6-month, 12-month, and 18-month PFS **(B)**.

The results of the multivariate Cox analysis of the training cohort indicated that ECOG PS, PD-L1 expression, chemotherapy cycle, and pretreatment LAR were independent prognostic factors affecting OS. Therefore, we incorporated these four factors into the construction of the nomogram model to predict the 6-month, 12-month, and 18-month OS (**Figure 2A**). Moreover, ECOG PS, histology, PD-L1 expression, chemotherapy cycle, pretreatment PLR, and pretreatment LAR were independent prognostic factors affecting PFS. Subsequently, we combined these six factors into the construction of the nomogram model to predict the 6-month, 12-month, and 18-month PFS (**Figure 2B**).

### 3.7 Validation of the nomogram

**Figure 3:**
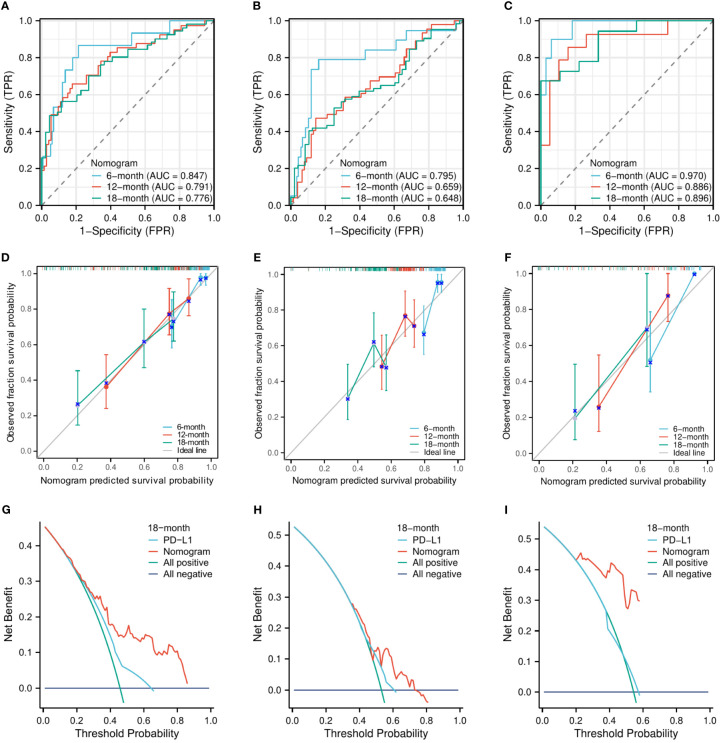
The evaluation of the nomogram for predicting OS. The area under the time-dependent receiver operating characteristic curves for predicting the 6-month, 12-month, and 18-month OS in the training cohort **(A)**, the internal validation cohort **(B)**, and the external validation cohort **(C)**. Calibration curves for predicting the 6-month, 12-month, and 24-month OS in the training cohort **(D)**, the internal validation cohort **(E)**, and the external validation cohort **(F)**. Decision curve for predicting the 18-month OS in the training cohort **(G)**, the internal validation cohort **(H)**, and the external validation cohort **(I)**.

The C-indexes of the nomogram model for predicting OS were 0.750 (95% CI, 0.718–0.783) in the training cohort, 0.684 (95% CI, 0.651–0.718) in the internal validation cohort, and 0.880 (95% CI, 0.843–0.917) in the external validation cohort. The AUCs for predicting the 6-month, 12-month, and 18-month OS were 0.847, 0.791, and 0.776, respectively, in the training cohort (**Figure 3A**); 0.795, 0.659, and 0.648, respectively, in the internal validation cohort (**Figure 3B**); and 0.970, 0.886, and 0.896, respectively, in the external validation cohort (**Figure 3C**). The calibration curves for the 6-month, 12-month, and 18-month OS probabilities showed a good agreement between predictions and actual observations (**Figures 3D–F**). In addition, the DCA curves of the nomogram model in predicting the 18-month OS showed good net benefits (**Figures 3G–I**).

**Figure 4:**
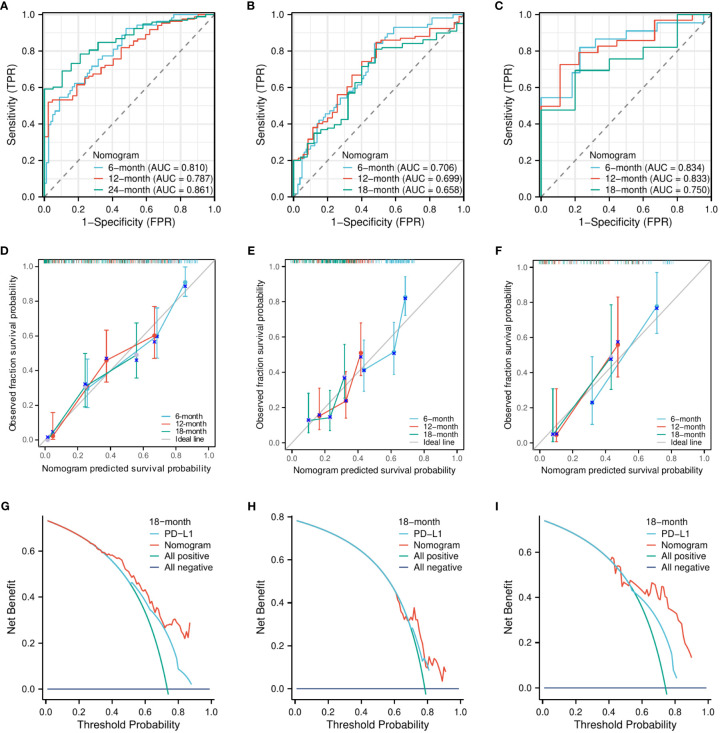
The evaluation of the nomogram for predicting PFS. The area under the time-dependent receiver operating characteristic curves for predicting the 6-month, 12-month, and 18-month PFS in the training cohort **(A)**, the internal validation cohort **(B)**, and the external validation cohort **(C)**. Calibration curves for predicting the 6-month, 12-month, and 24-month PFS in the training cohort **(D)**, the internal validation cohort **(E)**, and the external validation cohort **(F)**. Decision curve for predicting the 18-month PFS in the training cohort **(G)**, the internal validation cohort **(H)**, and the external validation cohort **(I)**.

Similarly, the C-indexes of the nomogram model for predicting PFS were 0.747 (95% CI, 0.723–0.771) in the training cohort, 0.665 (95% CI, 0.638–0.693) in the internal validation cohort, and 0.758 (95% CI, 0.716–0.800) in the external validation cohort. The AUCs for predicting the 6-month, 12-month, and 18-month OS were 0.810, 0.787, and 0.861, respectively, in the training cohort (**Figure 4A**); 0.706, 0.699, and 0.658, respectively, in the internal validation cohort (**Figure 4B**); and 0.834, 0.833, and 0.750, respectively, in the external validation cohort (**Figure 4C**). The calibration curves for the 6-month, 12-month, and 18-month OS probabilities showed a good agreement between predictions and actual observations (**Figures 4D–F**). In addition, the DCA curves of the nomogram model in predicting the 18-month OS showed good net benefits (**Figures 4G–I**).

## 4 Discussion

The efficacy of anti-PD-1 monotherapy is limited in the setting of NSCLC. Consequently, combination treatment has received widespread attention. Preclinical studies have confirmed that anti-PD-1 plus chemotherapy can achieve interactive synergistic effects. Furthermore, multiple clinical studies have demonstrated improvement in patients’ prognosis with the use of anti-PD-1 plus chemotherapy (19, 20). In our study, patients who received anti-PD-1 plus chemotherapy with or without antiangiogenic therapy had a median OS and PFS of 19 months and 8 months, respectively, with an ORR of 38.9%, suggesting that this combination therapy has a favorable clinical benefit. However, the selection of the best population is still challenging. Therefore, our study included advanced NSCLC patients from two independent centers. The results suggested that ECOG PS, PD-L1 expression, chemotherapy cycle, and pretreatment LAR were independent prognostic factors for OS, while ECOG PS, histology, PD-L1 expression, chemotherapy cycle, pretreatment PLR, and pretreatment LAR were independent prognostic factors for PFS. Then, a novel nomogram was constructed based on these factors to predict OS and PFS. So far, there is no valid and reliable predicted model to predict the prognosis of advanced NSCLC receiving anti-PD-1 plus chemotherapy with or without antiangiogenic therapy. The nomogram models in our study were evaluated and validated to have good predictive performance.

Pretreatment LAR, a novel hematological marker, is derived from the combination of serum LDH and Alb levels. LAR was found to be associated with survival in many malignancies and may be an independent poor prognostic factor (21, 22). Currently, there are few reports on the value of pretreatment LAR in advanced NSCLC. Our previous study found that pretreatment LAR was significantly associated with survival for advanced EGFR-mutated NSCLC (23). In this study, we also demonstrated that pretreatment LAR was an independent prognostic factor affecting OS, PFS, and ORR in advanced NSCLC patients who received anti-PD-1 plus chemotherapy with or without antiangiogenic therapy. LDH is an enzyme that plays a role in glycolysis, converting pyruvate into lactate. Serum LDH reflects the degree of tumor load and hypoxia (24). The increase in tumor volume leads to increased tumor cell load and hypoxia, which subsequently enhances glycolytic metabolism and results in an elevation of LDH levels (25, 26). High LDH levels may lead to lactate production and acidification of the extracellular environment, thereby inhibiting the antitumor immune response (27). Clinical studies have demonstrated that patients with high LDH do not respond well to immunotherapy (28). Serum Alb reflects the nutritional status of tumor patients. Poor nutritional status is usually correlated with worse survival. A retrospective study included NSCLC patients who were treated with PD-1 inhibitors and showed that patients with high Alb (≥3.5 g/dl) had better ORR, PFS, and OS (29). A possible explanation is that patients with poor nutritional status have a poorly functioning immune system that does not activate immune cells to kill tumor cells. LAR comprehensively reflects the nutritional status and systemic inflammation by integrating serum LDH and Alb, which might be promising hematological biomarkers for advanced NSCLC receiving PD-1 inhibition plus chemotherapy with or without antiangiogenic therapy.

In addition, PLR is another common hematologic marker reflecting the platelet to lymphocyte ratio, which is an important component of the systemic inflammatory response. Studies have confirmed that PLR was associated with the efficacy of immunotherapy in multiple solid tumors (30, 31). A retrospective study showed that PLR ≥200 was related to worse OS (HR, 1.94; 95% CI, 1.29–2.94; *P* = 0.002) and PFS (HR, 1.894; 95% CI, 1.27–2.82; *P* = 0.002) (31). Russo et al. also found that patients had shorter PFS in the high PLR group (30). Similar to their results, our study demonstrated that pretreatment PLR was a poor prognostic factor, and patients with high PLR had poor PFS. Therefore, pretreatment PLR may also serve as a significant biomarker for advanced NSCLC patients undergoing PD-1 inhibition combined with chemotherapy, regardless of antiangiogenic therapy.

PD-L1 expression is currently the most widely reliable clinical predictor of response to ICIs. Several clinical trials have confirmed that a higher level of PD-L1 expression was associated with a more substantial benefit from ICIs (7–9). The KEYNOTE-042 study compared pembrolizumab with chemotherapy for advanced NSCLC. The results showed that OS was significantly longer in the pembrolizumab group than in the chemotherapy group and patients with higher PD-L1 expression had a lower risk of death (8). In our study, PD-L1 expression was an important prognostic indicator as well, and the benefit was particularly significant for patients with PD-L1 expression ≥50%. Nevertheless, PD-L1 expression also has some inherent flaws and does not fully predict the efficacy of immunotherapy accurately (32, 33). Therefore, it is necessary to combine multiple markers that might improve predictive accuracy.

ECOG PS is also an important prognostic factor. A meta-analysis that included 67 studies evaluated the efficacy and safety of ICIs in patients with ECOG PS ≥2. The results showed that ECOG PS predicted not only the prognosis but also the response to ICIs (34). In addition, a large study included 1,426 patients with advanced NSCLC and confirmed that patients with an ECOG PS of 2 had a lower median OS (35). In our study, the multivariate analysis result similarly suggested that ECOG PS was an independent prognostic factor for OS and PFS. The number of chemotherapy cycle is an essential factor affecting prognosis as well. Our study revealed a significant prolongation of OS and PFS in patients who received four or more cycles of chemotherapy. Therefore, we recommend at least four cycles of platinum-based chemotherapy if the patient can tolerate it.

The nomogram is a convenient and reliable predictive tool (36). More studies focus on creating a nomogram to predict the prognosis of NSCLC patients receiving PD-1/PD-L1 monotherapy. For example, Yuan et al. established a nomogram for predicting treatment response and prognosis in NSCLC patients who were treated with anti-PD-1 (37). Moreover, Botticelli et al. also established a prognostic nomogram based on three factors (liver and lung metastases and ECOG PS) for predicting survival in NSCLC patients undergoing nivolumab (38). However, to date, no studies have constructed nomogram models to accurately predict the prognosis of patients with advanced NSCLC receiving PD-1 antibody plus chemotherapy with or without antiangiogenic therapy. To the best of our knowledge, our study was the first to build a nomogram model based on clinical characteristics and hematological markers to predict survival. This nomogram model has been evaluated and validated to have good predictive ability, which is worthy to be promoted in the clinic and may help physicians to make clinical treatment decisions.

Our study also has some limitations. Firstly, our study is a retrospective study with some unavoidable bias. Secondly, our study has a proportion of patients with missing PD-L1 expression data, which may affect the predictive value of the study population. Finally, the data were obtained from two independent medical centers in the same city, and the sample size of external validation in this study was limited. Therefore, further large sample size, prospective, multicenter studies are needed to validate our model in the future.

## 5 Conclusion

Our study built a novel nomogram, which was validated to accurately predict the prognosis of advanced NSCLC after receiving anti-PD-1 plus chemotherapy with or without antiangiogenic therapy. The nomogram has been validated and is worth promoting in the clinical setting.

## Data availability statement

The raw data supporting the conclusions of this article will be made available by the authors, without undue reservation.

## Ethics statement

The studies involving humans were approved by the Ethics Committee Review Board of Fujian Medical University Union Hospital and Fujian Cancer Hospital. The studies were conducted in accordance with the local legislation and institutional requirements. The ethics committee/institutional review board waived the requirement of written informed consent for participation from the participants or the participants’ legal guardians/next of kin because of the retrospective nature of this study.

## Author contributions

YW: Conceptualization, Data curation, Methodology, Software, Writing – original draft, Writing – review & editing. CL: Conceptualization, Data curation, Methodology, Software, Writing – original draft, Writing – review & editing. ML: Data curation, Formal Analysis, Investigation, Visualization, Writing – review & editing. YH: Data curation, Formal Analysis, Investigation, Visualization, Writing – review & editing. BD: Data curation, Formal Analysis, Investigation, Visualization, Writing – review & editing. NY: Resources, Software, Validation, Writing – review & editing. YZ: Resources, Software, Validation, Writing – review & editing. XJ: Resources, Software, Validation, Writing – review & editing. JCL: Conceptualization, Supervision, Writing – review & editing. JHL: Conceptualization, Funding acquisition, Project administration, Supervision, Writing – review & editing.

## References

[1] Sung H Ferlay J Siegel RL Laversanne M Soerjomataram I Jemal A. Global cancer statistics 2020: GLOBOCAN estimates of incidence and mortality worldwide for 36 cancers in 185 countries. CA Cancer J Clin (2021) 71(3):209–49. doi: 10.3322/caac.21660 33538338

[2] Chen Z Fillmore CM Hammerman PS Kim CF Wong KK. Non-small-cell lung cancers: a heterogeneous set of diseases. Nat Rev Cancer (2014) 14(8):535–46. doi: 10.1038/nrc3775 PMC5712844 25056707

[3] Hanna N Johnson D Temin S Baker S Jr. Brahmer J Ellis PM. Systemic therapy for stage IV non-small-cell lung cancer: american society of clinical oncology clinical practice guideline update. J Clin Oncol (2017) 35(30):3484–515. doi: 10.1200/JCO.2017.74.6065 28806116

[4] Reck M Rabe KF. Precision diagnosis and treatment for advanced non-small-cell lung cancer. N Engl J Med (2017) 377(9):849–61. doi: 10.1056/NEJMra1703413 28854088

[5] Reck M Remon J Hellmann MD. First-line immunotherapy for non-small-cell lung cancer. J Clin Oncol (2022) 40(6):586–97. doi: 10.1200/JCO.21.01497 34985920

[6] Gandhi L Rodriguez-Abreu D Gadgeel S Esteban E Felip E De Angelis F. Pembrolizumab plus chemotherapy in metastatic non-small-cell lung cancer. N Engl J Med (2018) 378(22):2078–92. doi: 10.1056/NEJMoa1801005 29658856

[7] Reck M Rodriguez-Abreu D Robinson AG Hui R Csoszi T Fulop A. Pembrolizumab versus chemotherapy for PD-L1-positive non-small-cell lung cancer. N Engl J Med (2016) 375(19):1823–33. doi: 10.1056/NEJMoa1606774 27718847

[8] Mok TSK Wu Y-L Kudaba I Kowalski DM Cho BC Turna HZ. Pembrolizumab versus chemotherapy for previously untreated, PD-L1-expressing, locally advanced or metastatic non-small-cell lung cancer (KEYNOTE-042): a randomised, open-label, controlled, phase 3 trial. Lancet (2019) 393(10183):1819–30. doi: 10.1016/S0140-6736(18)32409-7 30955977

[9] Herbst RS Baas P Kim DW Felip E Perez-Gracia JL Han JY. Pembrolizumab versus docetaxel for previously treated, PD-L1-positive, advanced non-small-cell lung cancer (KEYNOTE-010): a randomised controlled trial. Lancet (2016) 387(10027):1540–50. doi: 10.1016/S0140-6736(15)01281-7 26712084

[10] Sui H Ma N Wang Y Li H Liu X Su Y. Anti-PD-1/PD-L1 therapy for non-small-cell lung cancer: toward personalized medicine and combination strategies. J Immunol Res (2018) 2018:6984948. doi: 10.1155/2018/6984948 30159341 PMC6109480

[11] Doroshow DB Herbst RS. Treatment of advanced non-small cell lung cancer in 2018. JAMA Oncol (2018) 4(4):569–70. doi: 10.1001/jamaoncol.2017.5190 29494728

[12] Ramakrishnan R Huang C Cho HI Lloyd M Johnson J Ren X. Autophagy induced by conventional chemotherapy mediates tumor cell sensitivity to immunotherapy. Cancer Res (2012) 72(21):5483–93. doi: 10.1158/0008-5472.CAN-12-2236 PMC4577568 22942258

[13] Zhang L Dermawan K Jin M Liu R Zheng H Xu L. Differential impairment of regulatory T cells rather than effector T cells by paclitaxel-based chemotherapy. Clin Immunol (2008) 129(2):219–29. doi: 10.1016/j.clim.2008.07.013 18771959

[14] Leonetti A Wever B Mazzaschi G Assaraf YG Rolfo C Quaini F. Molecular basis and rationale for combining immune checkpoint inhibitors with chemotherapy in non-small cell lung cancer. Drug Resist Updat (2019) 46:100644. doi: 10.1016/j.drup.2019.100644 31585395

[15] Paz-Ares L Vicente D Tafreshi A Robinson A Soto Parra H Mazieres J. A randomized, placebo-controlled trial of pembrolizumab plus chemotherapy in patients with metastatic squamous NSCLC: protocol-specified final analysis of KEYNOTE-407. J Thorac Oncol (2020) 15(10):1657–69. doi: 10.1016/j.jtho.2020.06.015 32599071

[16] Rocco D Della Gravara L Battiloro C Gridelli C. The role of combination chemo-immunotherapy in advanced non-small cell lung cancer. Expert Rev Anticancer Ther (2019) 19(7):561–8. doi: 10.1080/14737140.2019.1631800 31188040

[17] Evans M O'Sullivan B Smith M Taniere P. Predictive markers for anti-PD-1/PD-L1 therapy in non-small cell lung cancer-where are we? Transl Lung Cancer Res (2018) 7(6):682–90. doi: 10.21037/tlcr.2018.06.09 PMC6249622 30505713

[18] Seymour L Bogaerts J Perrone A Ford R Schwartz LH Mandrekar S. iRECIST: guidelines for response criteria for use in trials testing immunotherapeutics. Lancet Oncol (2017) 18(3):e143–e52. doi: 10.1016/S1470-2045(17)30074-8 PMC5648544 28271869

[19] Paz-Ares L Luft A Vicente D Tafreshi A Gumus M Mazieres J. Pembrolizumab plus chemotherapy for squamous non-small-cell lung cancer. N Engl J Med (2018) 379(21):2040–51. doi: 10.1056/NEJMoa1810865 30280635

[20] Wang J Lu S Yu X Hu Y Sun Y Wang Z. Tislelizumab plus chemotherapy vs chemotherapy alone as first-line treatment for advanced squamous non-small-cell lung cancer: A phase 3 randomized clinical trial. JAMA Oncol (2021) 7(5):709–17. doi: 10.1001/jamaoncol.2021.0366 PMC8017481 33792623

[21] Peng R-R Liang Z-G Chen K-H Li L Qu S Zhu X-D. Nomogram based on lactate dehydrogenase-to-albumin ratio (LAR) and platelet-to-lymphocyte ratio (PLR) for predicting survival in nasopharyngeal carcinoma. J Inflammation Res (2021) 14:4019–33. doi: 10.2147/JIR.S322475 PMC8385134 34447260

[22] Xie Z Zhou H Wang L Wu Y. The Significance of the preoperative lactate dehydrogenase/albumin Ratio in the Prognosis of Colon Cancer: a retrospective study. PeerJ (2022) 10:e13091. doi: 10.7717/peerj.13091 35295561 PMC8919845

[23] Wu Y Du B Lv C Ji X Lai J. LAPS score for individualized treatment of advanced EGFR-mutated non-small cell lung cancer receiving EGFR-TKIs with or without bevacizumab. Ann Med (2023) 55(2):2257227. doi: 10.1080/07853890.2023.2257227 37713583 PMC10506427

[24] Smith H Board M Pellagatti A Turley H Boultwood J Callaghan R. The effects of severe hypoxia on glycolytic flux and enzyme activity in a model of solid tumors. J Cell Biochem (2016) 117(8):1890–901. doi: 10.1002/jcb.25488 26755257

[25] Semenza GL. Defining the role of hypoxia-inducible factor 1 in cancer biology and therapeutics. Oncogene (2010) 29(5):625–34. doi: 10.1038/onc.2009.441 PMC2969168 19946328

[26] Zha X Wang F Wang Y He S Jing Y Wu X. Lactate dehydrogenase B is critical for hyperactive mTOR-mediated tumorigenesis. Cancer Res (2011) 71(1):13–8. doi: 10.1158/0008-5472.CAN-10-1668 21199794

[27] Koukourakis MI Giatromanolaki A Sivridis E Bougioukas G Didilis V Gatter KC. Lactate dehydrogenase-5 (LDH-5) overexpression in non-small-cell lung cancer tissues is linked to tumour hypoxia, angiogenic factor production and poor prognosis. Br J Cancer (2003) 89(5):877–85. doi: 10.1038/sj.bjc.6601205 PMC2394471 12942121

[28] Mezquita L Auclin E Ferrara R Charrier M Remon J Planchard D. Association of the lung immune prognostic index with immune checkpoint inhibitor outcomes in patients with advanced non-small cell lung cancer. JAMA Oncol (2018) 4(3):351–7. doi: 10.1001/jamaoncol.2017.4771 PMC5885829 29327044

[29] Pu D Xu Q Zhou LY Zhou YW Liu JY Ma XL. Inflammation-nutritional markers of peripheral blood could predict survival in advanced non-small-cell lung cancer patients treated with PD-1 inhibitors. Thorac Cancer (2021) 12(21):2914–23. doi: 10.1111/1759-7714.14152 PMC8563162 34581010

[30] Russo A Russano M FranChina T Migliorino MR Aprile G Mansueto G. Neutrophil-to-lymphocyte ratio (NLR), platelet-to-lymphocyte ratio (PLR), and outcomes with nivolumab in pretreated non-small cell lung cancer (NSCLC): A large retrospective multicenter study. Adv Ther (2020) 37(3):1145–55. doi: 10.1007/s12325-020-01229-w 32002809

[31] Kartolo A Holstead R Khalid S Emack J Hopman W Robinson A. Serum neutrophil-to-lymphocyte ratio and platelet-to-lymphocyte ratio in prognosticating immunotherapy efficacy. Immunotherapy (2020) 12(11):785–98. doi: 10.2217/imt-2020-0105 32657234

[32] Hirsch FR McElhinny A Stanforth D Ranger-Moore J Jansson M Kulangara K. PD-L1 immunohistochemistry assays for lung cancer: results from phase 1 of the blueprint PD-L1 IHC assay comparison project. J Thorac Oncol (2017) 12(2):208–22. doi: 10.1016/j.jtho.2016.11.2228 27913228

[33] McLaughlin J Han G Schalper KA Carvajal-Hausdorf D Pelekanou V Rehman J. Quantitative assessment of the heterogeneity of PD-L1 expression in non-small-cell lung cancer. JAMA Oncol (2016) 2(1):46–54. doi: 10.1001/jamaoncol.2015.3638 26562159 PMC4941982

[34] Tomasik B Bienkowski M Braun M Popat S Dziadziuszko R. Effectiveness and safety of immunotherapy in NSCLC patients with ECOG PS score >/=2 - Systematic review and meta-analysis. Lung Cancer (2021) 158:97–106. doi: 10.1016/j.lungcan.2021.06.004 34144405

[35] Spigel DR McCleod M Jotte RM Einhorn L Horn L Waterhouse DM. Safety, efficacy, and patient-reported health-related quality of life and symptom burden with nivolumab in patients with advanced non-small cell lung cancer, including patients aged 70 years or older or with poor performance status (CheckMate 153). J Thorac Oncol (2019) 14(9):1628–39. doi: 10.1016/j.jtho.2019.05.010 31121324

[36] Iasonos A Schrag D Raj GV Panageas KS. How to build and interpret a nomogram for cancer prognosis. J Clin Oncol (2008) 26(8):1364–70. doi: 10.1200/JCO.2007.12.9791 18323559

[37] Yuan S Xia Y Shen L Ye L Li L Chen L. Development of nomograms to predict therapeutic response and prognosis of non-small cell lung cancer patients treated with anti-PD-1 antibody. Cancer Immunol Immunother (2021) 70(2):533–46. doi: 10.1007/s00262-020-02710-9 PMC10992866 32852602

[38] Botticelli A Salati M Di Pietro FR Strigari L Cerbelli B Zizzari IG. A nomogram to predict survival in non-small cell lung cancer patients treated with nivolumab. J Transl Med (2019) 17(1):99. doi: 10.1186/s12967-019-1847-x 30917841 PMC6437908

